# CD4-independent use of the CCR5 receptor by sequential primary SIVsm isolates

**DOI:** 10.1186/1742-4690-4-50

**Published:** 2007-07-23

**Authors:** Anna Laurén, Elzbieta Vincic, Hiroo Hoshino, Rigmor Thorstensson, Eva Maria Fenyö

**Affiliations:** 1 Department of Laboratory Medicine, Division of Medical Microbiology/Virology, Lund University, Lund, Sweden; 2 Department of Virology and Preventive Medicine, Gunma University Graduate School of Medicine, Gunma, Japan; 3 Swedish Institute for Infectious Disease Control, Stockholm, Sweden

## Abstract

**Background:**

CD4-independence has been taken as a sign of a more open envelope structure that is more accessible to neutralizing antibodies and may confer altered cell tropism. In the present study, we analyzed SIVsm isolates for CD4-independent use of CCR5, mode of CCR5-use and macrophage tropism. The isolates have been collected sequentially from 13 experimentally infected cynomolgus macaques and have previously been shown to use CCR5 together with CD4. Furthermore, viruses obtained early after infection were neutralization sensitive, while neutralization resistance appeared already three months after infection in monkeys with progressive immunodeficiency.

**Results:**

Depending whether isolated early or late in infection, two phenotypes of CD4-independent use of CCR5 could be observed. The inoculum virus (SIVsm isolate SMM-3) and reisolates obtained early in infection often showed a pronounced CD4-independence since virus production and/or syncytia induction could be detected directly in NP-2 cells expressing CCR5 but not CD4 (CD4-independent-HIGH). Conversely, late isolates were often more CD4-dependent in that productive infection in NP-2/CCR5 cells was in most cases weak and was revealed only after cocultivation of infected NP-2/CCR5 cells with peripheral blood mononuclear cells (CD4-independent-LOW). Considering neutralization sensitivity of these isolates, newly infected macaques often harbored virus populations with a CD4-independent-HIGH and neutralization sensitive phenotype that changed to a CD4-independent-LOW and neutralization resistant virus population in the course of infection. Phenotype changes occurred faster in progressor than long-term non-progressor macaques. The phenotypes were not reflected by macrophage tropism, since all isolates replicated efficiently in macrophages. Infection of cells expressing CCR5/CXCR4 chimeric receptors revealed that SIVsm used the CCR5 receptor in a different mode than HIV-1.

**Conclusion:**

Our results show that SIVsm isolates use CCR5 independently of CD4. While the degree of CD4 independence and neutralization sensitivity vary over time, the ability to productively infect monocyte-derived macrophages remains at a steady high level throughout infection. The mode of CCR5 use differs between SIVsm and HIV-1, SIVsm appears to be more flexible than HIV-1 in its receptor requirement. We suggest that the mode of CCR5 coreceptor use and CD4-independence are interrelated properties.

## Background

Human immunodeficiency virus (HIV) and the simian counterpart, simian immunodeficiency virus (SIV) normally enter and infect cells after engagement with CD4 and a coreceptor, usually a chemokine receptor (reviewed by [1]). Binding to CD4 induces a conformational change to form and expose the coreceptor binding site. Further binding to the coreceptor induces additional rearrangements such that fusion between the viral envelope and the cell membrane can take place. The two major coreceptors used by HIV type-1 (HIV-1) are CCR5 and CXCR4 [2-4]. Viruses using the CCR5 coreceptor (R5-phenotype) predominate early in asymptomatic HIV-1 infection, while CXCR4-using HIV-1 (X4 phenotype) can be isolated in approximately half of the patients that progress to AIDS [5,6]. CCR5 is also the major coreceptor for SIV [7-9]. Furthermore, both HIV and SIV have been shown to use a wide set of alternative coreceptors, including CCR1, CCR2b, CCR3, CXCR6, CCR8, CX3CR1/V28, gpr1, gpr15, APJ, ChemR23 and RDC1, but the *in vivo *role of these coreceptors is still unknown [1].

CD4-independent use of coreceptors by HIV and SIV has long been an intriguing question. It has been assumed that CD4-independence is a sign of a more open envelope structure, more accessible to neutralizing antibodies and conferring altered cell tropism. HIV type 2 (HIV-2) and some SIV strains have been shown to enter cells independently of CD4 [10-14]. This was followed by reports on laboratory adapted HIV-1 variants that were able of CD4-independent infections [15-18]. CD4-independent HIV-1 was found to have a stable exposure of the coreceptor binding site [17]. However, similar conformational changes in the envelope have not yet been shown for SIV or HIV-2 and it may be that the HIV-1 envelope is more dependent on conformational changes for efficient infection than HIV-2 and SIV. Primary HIV-1 isolates that can infect cells independently of CD4 are rare and have not been isolated until lately [19]. However, Gorry *et al. *described a neurovirulent macrophage-tropic HIV-1 isolate that had increased affinity for CCR5 and could infect cells at minimal levels of CD4 [20]. Likewise, CD4-independence of SIV envelopes has been correlated to macrophage tropism and sensitivity to neutralization by heterologous sera or monoclonal antibodies [21,22]. Similar association between CD4-independent cell entry and sensitivity to neutralization has been reported for HIV-1 and HIV-2 [23-26]. Nevertheless, a thorough study of the relationships between macrophage tropism, neutralization sensitivity and CD4-independence of a large number of primary virus isolates has not yet been performed.

Our previous studies on CD4-independent use of CCR5 and gpr15 by envelopes of sequential SIVsm isolates (of sooty mangabey origin) showed that early reisolates from macaques infected with a CD4-independent inoculum maintained envelopes with a broad range of CD4-independent use of CCR5 in a fusion assay [27]. Envelopes from late reisolates at the time when the macaques had developed neutralizing antibodies were CD4-dependent. Infection with a CD4-dependent virus resulted in evolution to CD4-independence in late reisolates, indicating that CD4-dependent use of coreceptors may change in the course of infection [27]. Similarly, in two other studies, rapid progression of simian AIDS was accompanied by selection for CD4-independent variants [28,29]. Rapid disease was characterized by absent or transient humoral and cellular immune responses, high levels of virus replication and widespread dissemination of SIV in macrophages and multinucleated cells [28]. These studies did not, however, investigate macaques with a slow disease progression. Neither was neutralization sensitivity or macrophage tropism of the virus variants studied. This prompted us to investigate these issues in our material consisting of 13 cynomolgus macaques with different disease patterns. Sequential SIVsm isolates that previously have been characterized for coreceptor use and neutralization sensitivity were available [30,31]. All isolates, but one, used CCR5 for cell entry, and CCR5 was also the major coreceptor in 70 out of 105 isolates tested. Macrophage tropism, evaluated as relative replication capacity (relative to replication of SIVmac251) in monocyte-derived macrophages, coreceptor use and sensitivity to neutralization by autologous and heterologous sera, varied with severity of SIVsm infection. Long-term non-progressor (LTNP) macaques appeared to control virus in that virus isolates, if obtained at all, showed limited ability to use coreceptors late in infection [31]. On the other hand, reisolates from the majority of macaques with progressive disease maintained use of a wide variety of coreceptors and an effective replication capacity in macrophages throughout the 1–5 years study period. Furthermore, neutralization resistant variants emerged earlier in progressor macaques than in LTNP macaques [30]. In the present study we further analyse these isolates, focusing on CD4-independence, the mode of CCR5-use and macrophage tropism. We show that CD4-independent use of CCR5 and macrophage tropism are general properties of primary SIVsm isolates obtained from animals infected with a CD4-independent virus. CD4-independence is more pronounced early in infection than late. Phenotypic changes, like an increase in dependence on CD4 and neutralization resistance seem to occur earlier in progressor (P) and slow-progressor (SP) macaques than in LTNP animals while replication capacity in macrophages did not change during pathogenesis.

## Results

### CD4-independent infections of NP-2 cells

NP-2 cells expressing both CD4 and CCR5 were readily infected by CCR5-using (R5) SIVsm reisolates derived from virus isolation cultures with peripheral blood mononuclear cells (PBMC) of either macaque origin (mPBMC, 11 isolates) or human origin (hPBMC, 44 isolates) (data not shown). All isolates but one were previously shown to use CCR5 when tested on GHOST(3)-CCR5 and U87.CD4-CCR5 cells [31]. One isolate derived by cocultivation on hPBMC used CXCR4 and CXCR6 only (12-month isolate from macaque D24) and could not infect NP-2 cells expressing CD4 and CCR5. Clinical status of the macaques and description of the virus isolates are supplemented [see Additional file 1].

**Figure 1:**
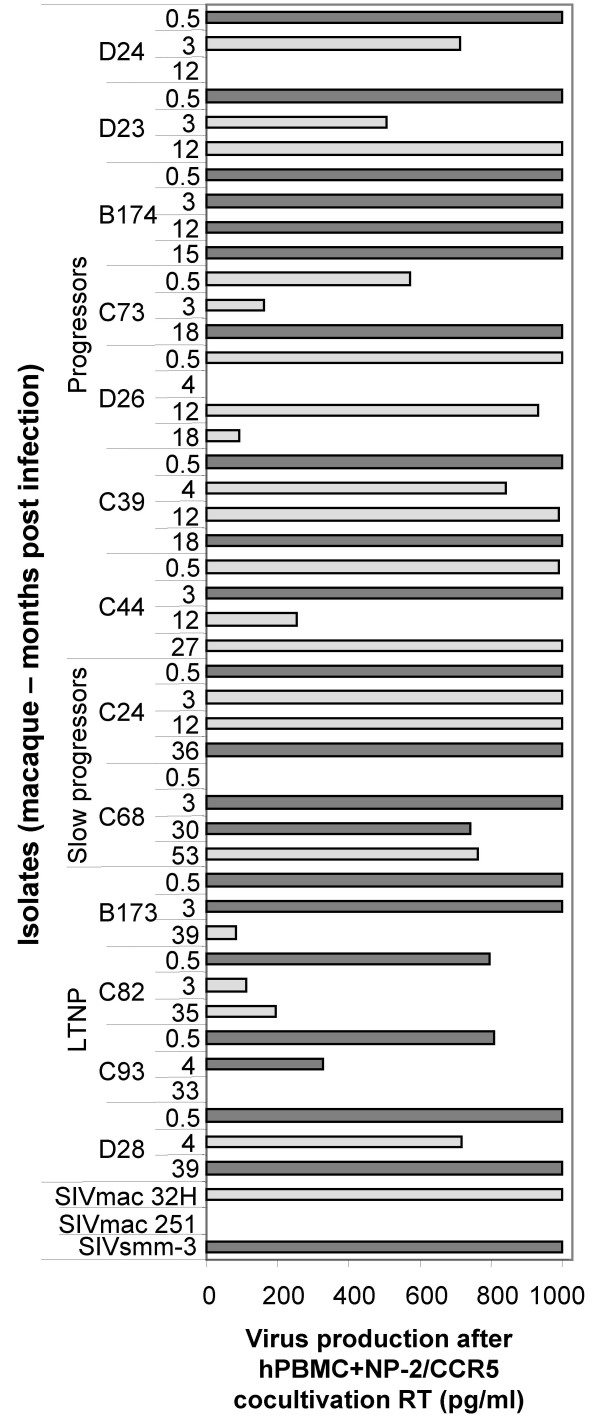
**CD4-independent use of CCR5 by isolates obtained on hPBMC**. NP-2/CCR5 cells were infected with virus stocks containing 2.7–3.5 log10 pg RT/well. The day after infection cultures were washed extensively and fresh medium was added. Infected NP-2/CCR5 cells were followed for syncytia induction up to seven days after infection. RT was analyzed in supernatants from NP-2 cells at day 1 after wash and before start of cocultivation. Cocultivation of NP-2/CCR5 cells with hPBMC was started seven days after infection and virus production was measured after additional 6 days. CD4-independent-HIGH, virus production and/or syncytia induction could be detected directly in NP-2/CCR5 cells (dark grey). CD4-independent-LOW, productive infection in NP-2/CCR5 cells revealed only after cocultivation of infected NP-2/CCR5 cells with hPBMC (light grey). RT was analyzed with undiluted supernatants and therefore values above 1000 pg RT/ml cannot be separated. Detection limit for RT was 50 pg/ml. Values are means of duplicate infections.

CD4-independent use of CCR5 was tested in two ways. First, NP2/CCR5 cells were infected and culture supernatants tested for reverse transcriptase (RT) activity and cultures observed for syncytia formation. Isolates which were positive using one or both of these parameters were defined as the phenotype CD4-independent-HIGH. However, it is possible that the absence of CD4 might reduce the amount of syncytia and therefore it was important to analyse infection by additional techniques. To explore whether the virus production and syncytia negative NP-2/CCR5 cultures were infected at all, we followed a second strategy. Infected cultures were trypsinized 7 days after infection, cells were washed once with PBS and added to new culture plates together with PHA-P stimulated hPBMC. Another six days later, supernatants were collected and analyzed for reverse transcriptase activity. The phenotype of isolates that were positive for CD4-independent use of CCR5 only after coculture with PBMC was defined as CD4-independent-LOW. Surprisingly, the SIVmac 32H isolate, known to use CCR5 independently of CD4, was of CD4-independent-LOW phenotype (Figure 1). Our results showed that, indeed, a majority of viruses were able to use CCR5 independently of CD4 to enter cells. However, infection of cells expressing CCR5 together with CD4 was at all times more effective than infection of cells expressing only CCR5.

Comparison of isolates obtained on monkey and human PBMC showed that viruses isolated on mPBMC had more often CD4-independent-HIGH phenotype than viruses isolated on hPBMC. In fact, the majority (seven out of eleven) of viruses isolated on mPBMC was of CD4-independent-HIGH phenotype and induced both syncytia and virus production in the NP-2/CCR5 cells (Table 1). The remaining four isolates obtained on mPBMC appeared to be CD4-independent-LOW. Isolation on hPBMC distinguished these phenotypes in a time-dependent manner (Figure 1). Accordingly, early isolates (defined as 2-week and 3 or 4-month isolates) from 11 macaques out of 13 and late isolates (defined as 12-month and/or later isolates) from only five macaques (out of 13) were able to induce syncytia and/or produce RT in NP-2/CCR5 cells (CD4-independent HIGH). However, when considering the difference between the two matched proportions (sequential early and late isolates) the difference was not statistically significant (p = 0.0703, two sided McNemar's test). McNemar's test measures the number of changes comparing matched early and late time-points and significant proportions were not reached due to the small number of samples. Phenotypes of sequential isolates changed in six animals from CD4-independent-HIGH to CD4-independent-LOW, while isolates from four animals appeared CD4-independent-HIGH both early and late in infection. Change in phenotype from CD4-independent-LOW to CD4-independent-HIGH was only observed in one animal and in two macaques the CD4-independent-LOW phenotype did not seem to change throughout infection.

**Table 1:** Comparison of the capacity to infect NP-2 cells by isolates on mPBMC or hPBMC.

**Origin of cells for virus isolation**	**Isolate^a^**			**NP-2/CD4/CCR5 syncytia^b^**	**NP-2/CCR5 syncytia**	**NP-2/CCR5+PBMC virus production ^c ^RT (pg/ml)**
				
	**Monkey**	**Time PI (months)**				
mPBMC	D24	0.5		++++	+++	>1000
		3		++++	+	803
		10	§	+++	-	560
	C73	5	§	++++	++	>1000
		7	§	++++	++	649
		18		++++	+	450
	C68	0.5	#	++	-	526
		30	#	++	-	674
		53	#	++	-	78
	B173	0.5		++++	+++	998
		39		++++	+	827
hPBMC	D24	0.5		+++	-	>1000
		3		++	-	713
		12	§	-	-	<50
	C73	0.5	§	++	-	573
		3	§	++	-	161
		18		++++	-	>1000
	C68	0.5		++++	-	<50
		3	§	++++	-	>1000
		30		++++	-	741
		53		++++	-	762
	B173	0.5		++++	+/-	>1000
		90	§	++	-	>1000
		39		+	-	85

^a ^Cells were infected with virus stocks containing 2.7–3.5 log10 pg RT/well except for indicated (#) isolates that were infected with 1.9–2.3 log10 pg RT/well. §Isolates that could not be obtained on corresponding time-points when isolating viruses on mPBMC and hPBMC, respectively. PI, time for virus isolation post infection.

^b^ Induction of syncytia was observed in light microscope 5 and 7 days after infection. -, no syncytia; +, 10–20 syncytia per well; ++, syncytia covering 20–50% of the wells; +++, syncytia covering 50–90% of the wells; ++++, syncytia covering >90% of the wells

^c ^Virus production was measured six days after start of cocultures with human PBMC (hPBMC). Values are means of two independent infections in duplicate wells. Supernatant culture fluids were collected at day 7 and production of RT was analyzed. Supernatants were undiluted in the RT assay and therefore values above 1000 pg RT/ml cannot be separated. Cut-off detection level was 50 pg RT/ml

Considering changes of CD4-independence in relation to disease progression, phenotypic changes seemed to occur slower in LTNP macaques than in P and SP macaques. As early as two-weeks after infection four out of nine macaques with progressive disease harbored viruses that had changed to CD4-independent-LOW. In contrast, virus isolated from the four LTNP macaques was of the CD4-independent-HIGH phenotype, the same phenotype as that of the inoculum virus. At three or four months after infection CD4-independent-HIGH viruses were still isolated in two out of four LTNP animals, while only three out of nine macaques from the P and SP group harbored viruses with the CD4-independent-HIGH phenotype. CD4-independence of the P and SP group was fluctuating and at the last time point four out nine macaques were CD4-independent-HIGH. In contrast, in LTNP macaques virus evolution was narrowed further and the CD4-independent-HIGH phenotype was only apparent in one out of four macaques.

### Intracellular and extracellular virus maturation

**Figure 2:**
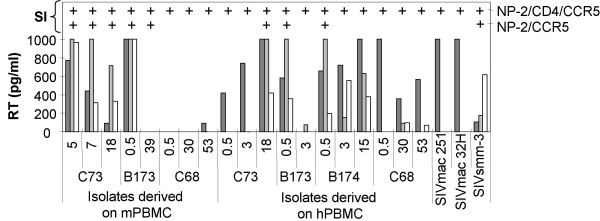
**Intracellular and extracellular virus maturation shown by infection of hPBMC with lysates and supernatants of NP-2/CCR5 cultures**. Seven days after infection, NP-2 cells were trypsinized, washed with PBS and lysed by 0.001% Triton X-100 followed by one cycle of freeze-thawing step. Lysates were titrated at five-fold dilution steps on hPBMC. Supernatant culture fluids from hPBMC infections were collected at day 7 and production of RT was analyzed with undiluted supernatants. The RT cut-off detection level was 50 pg/ml and values above 1000 pg/ml could not be separated. Dark grey bars represent mean virus production in NP-2/CD4/CCR5 cells and. light grey bars represent virus production in NP-2/CCR5 cells. White bars represent virus production measured by RT in PBMC infected with cell lysates diluted 1:5 from infected NP-2/CCR5 cells. Positive syncytia induction (SI) are indicated with +. Means of RT production in duplicates of infection are indicated.

HIV-1 virions can assemble and mature intracellularly within macrophages and retain infectivity for several weeks [32]. This prompted us to test whether viruses had matured intracellularly in the NP-2/CCR5 cells and upon cocultivation transferred to hPBMC in a cell-to-cell fashion. To study possible intracellular virus assembly and maturation, the infected NP-2 cells were detached with trypsin and washed with PBS, lysed by the addition of 0.001% Triton X-100 and one cycle of freeze-thawing. Visual inspection in the light microscope and culturing attempts showed that cell lysis was complete. The lysates were then titrated on hPBMC. Presence of intracellular virus was demonstrated in infections of NP-2/CCR5 cells with 11 out of 19 isolates tested (Figure 2). It is possible that the lysis procedure affected the infectivity of viruses and this could account for the negative cultures. Interestingly, the majority of virus isolates that established infection in hPBMC after infection with lysed cells were of the CD4-independent-HIGH phenotype. Similar lysis experiments were not performed with NP-2/CD4/CCR5 cell lines because these infections showed stronger cytopathic effect (large syncytia and pronounced cell death) than NP-2/CCR5 cultures.

### Mode of CCR5 use

**Figure 3:**
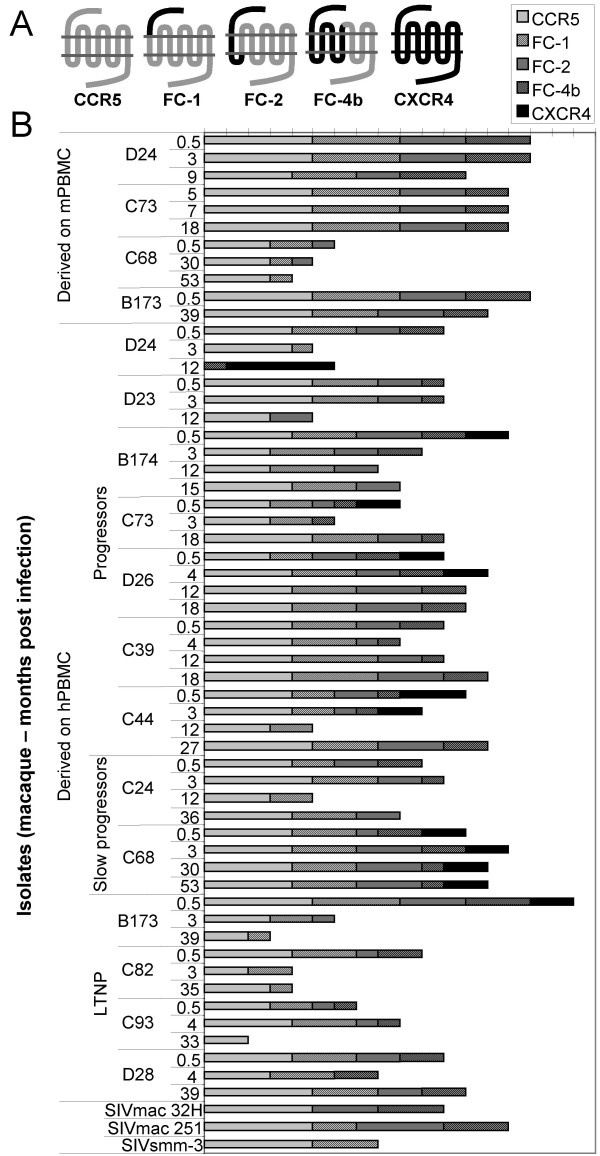
**Mode of CCR5-use**. A. Schematic figure of CCR5 and the chimeric receptors FC-1, FC-2 and FC-4b. The CCR5 part is represented as grey and the CXCR4 part as black. B. Mode of CCR5-use was analyzed by infection of U87.CD4 cells expressing CCR5 or chimeric receptors. Length of bars indicates degree of infection and syncytia induction as observed in a light microscope 5 and 7 days after infection. Degree of infection follows a scale from 0 to 5 where 0 is no syncytia and RT negative; 1 was <10 syncytia per well or RT positive; 2 was 10–20 syncytia per well; 3 indicates syncytia covering 20–50% of the wells; 4 indicates syncytia covering 50–90% of the wells; 5 indicates syncytia covering >90% of the wells. RT production was positive in grades ranging from 2 to 5 and in concordance with syncytia induction.

To further dissect CCR5-use by SIV, the mode of CCR5-use was evaluated in U87.CD4 cell lines expressing chimeric receptors constructed of CCR5 and CXCR4 (Figure 3) [33]. In the present experiments we used three chimeras (FC-1, FC-2 and FC-4b), in which CCR5 had been exchanged gradually, beginning with the N-terminal, for corresponding parts of the CXCR4 molecule. FC-1 and FC-2 differ in the first transmembrane portion, which is CCR5 in FC-1 and CXCR4 in the FC-2 chimera. The CXCR4 portion of FC-4b extends to the fourth transmembrane region. The U87.CD4 cell line is known to endogenously express other SIV coreceptors (GPR1 and CXCR6) known to be used by SIV [34,35] and to control for possible GPR1 or CXCR6 use, the U87.CD4 parental cell line was included in all experiments. No syncytia induction or viral antigen production was observed in the U87.CD4 parental cells in parallel infections with the SIVsm isolates (data not shown). Our results showed that the FC-1 receptor was frequently used by SIVsm (93% of the hPBMC reisolates) and FC-2 and FC-4b were also used by a high number of isolates (78% and 71% of hPBMC reisolates, respectively, Figure 3). However use of FC-2 and FC-4b was rarely as effective as FC-1 use. Interestingly, the 12-month isolates from macaque D24 which has an unusual X4X6 phenotype [31] was only able to use FC-4b among the panel of chimeric receptors used in this study. Twenty-nine out of 45 of the hPBMC reisolates and eight out of 11 of the mPBMC reisolates could use all three chimeric receptors. There was no relationship between the isolates capacity to infect cells with the different chimeric receptors and disease progression of the animals.

### Replication in human and macaque MDM

**Figure 4:**
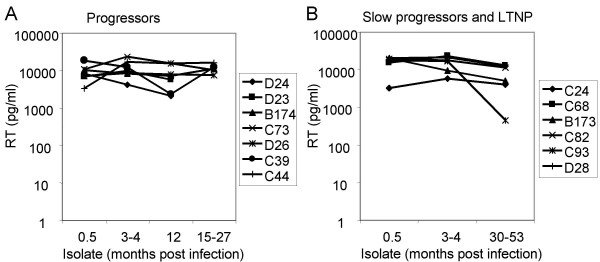
**Replication capacity in macrophages of viruses isolated on hPBMC from progressors (A) versus slow progressors and LTNP (B)**. Macrophages were infected with virus stocks containing 3.0–3.1 log10 pg RT/well (in 88% of the cultures) and RT production was measured in MDM 15 days after infection. Values are means from at least two experiments with MDM from different donors.

Reisolates from all monkeys (45 isolates derived on hPBMC) could readily infect human MDM (Figure 4). The majority of isolates replicated efficiently and showed high virus production in supernatants 15 days after infection (values above 5000 pg/ml). Also the SIVsm isolate SMM-3 that was used to infect the macaques replicated efficiently (14111 pg RT/ml). A few isolates showed lower replication efficacy (range 460 to 4185 pg RT/ml) and these isolates also widely varied in replication in MDM from different blood donors. A comparison between five virus isolates obtained on monkey as well as human PBMC showed similar replication capacities in monkey MDM (data not shown). Performing the same experiment on MDM of human origin showed that isolates obtained on macaque PBMC tended to replicate to lower levels (range 595 to 2100 pg RT/ml) relative to isolates obtained on human PBMC (range 1770 to 23732 pg RT/ml). Nevertheless, the hierarchy of replication capacities among isolates was the same (data not shown). Due to the limited availability of macaque blood we could not perform all experiments with cells from macaque origin.

We also evaluated replication capacity in macrophages relative to the first isolate of each macaque as an alternative to observe changes in macrophage-tropism over-time. We found that the relative differences for macrophage-tropism when comparing early and late isolates was in the range of 1 to 4.8-fold as measured by virus production in macrophages day 15 (with one exception (monkey C93) the difference was 28-fold). However, changes in macrophage tropism could not be correlated to progression of disease.

### CD4-independent use of CCR5 and neutralization sensitivity of SIVsm isolates

**Figure 5:**
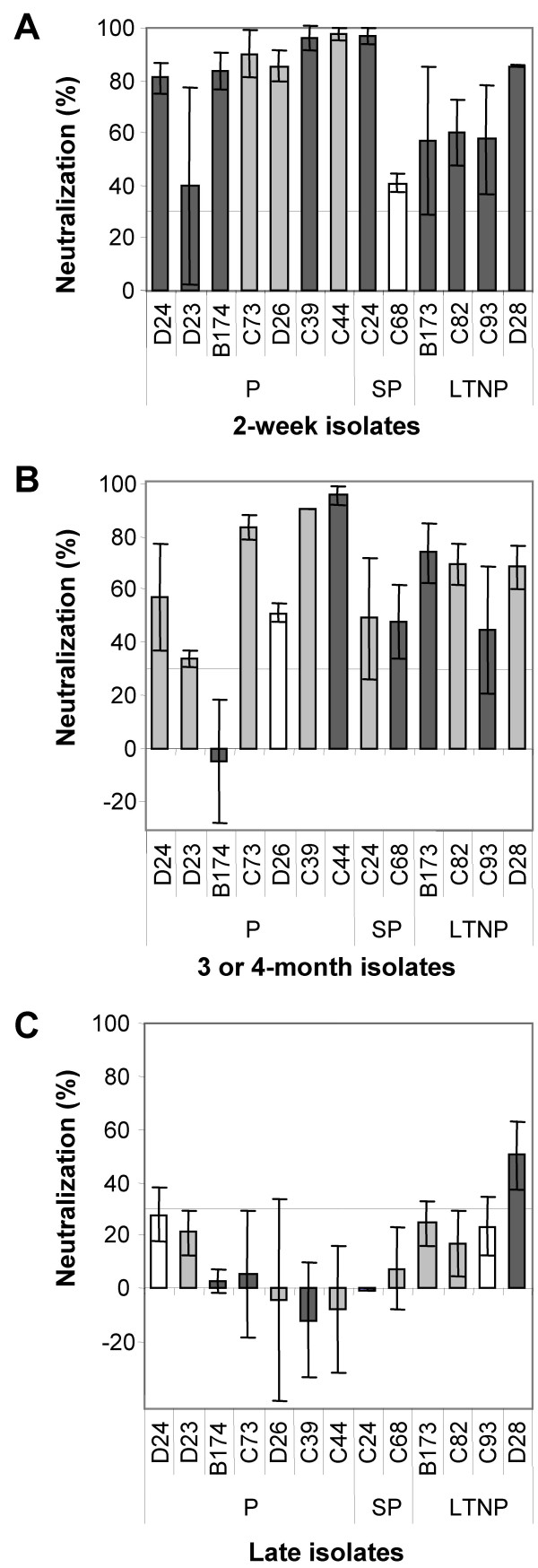
**CD4-independence and neutralization sensitivity**. Phenotypic changes in CD4-independence and sensitivity of neutralization over-time in 13 macaques. Neutralization sensitivity of three isolates (A, 2-week isolates, B, 3 or 4-month isolates and C, late isolates) from each macaque was tested with 1:20 dilution of serum [30]. Neutralization was also performed with autologous serum which gave similar results (data not shown). Neutralization sensitivity was measured using the GHOST(3) cell plaque reduction assay which has a cut-off for neutralization at 30% (marked with a line), that is results below 30% are negative [67]. The majority of newly infected macaques harbored virus populations with a CD4-independent-HIGH (dark grey bars) and neutralization sensitive phenotype. This phenotype gradually changed to become a CD4-independent-LOW (light grey bars) and neutralization resistant (below 30%) virus population. CD4-dependent isolates (white bars) were seen in both neutralization sensitive and neutralization resistant populations. Values are mean neutralization (+/- SD) of two independent assays performed in triplicates.

We asked the question if CD4-independence of the SIVsm reisolates evaluated in this study correlated with neutralization sensitivity, evaluated previously [30]. For this purpose we compared CD4-independence and neutralization sensitivity evaluated as neutralization by a high titer serum (H55:16) from a LTNP macaque (Figure 5). Similar changes in neutralization sensitivity were also evident when tested by autologous sera [30]. Two parameters, CD4-independence and neutralization sensitivity allowed us to examine early and late isolates for phenotypic changes. The results show that early in infection CD4-independent-HIGH and neutralization sensitive populations were in majority, since reisolates from all four LTNP and five out of nine SP/P macaques showed this phenotype. A few months later this population decreased (two out of nine P/SP macaques and two out of four LTNP macaques) and virus populations with CD4-independent-LOW and neutralization sensitive phenotypes expanded. In one P animal a CD4-independent-HIGH and neutralization resistant phenotype appeared already three months after infection and this population became prevalent at late stages in the P/SP group.

## Discussion

Sequential SIVsm reisolates from 13 macaques with different disease progression were monitored for CD4-independent use of CCR5, mode of CCR5-use and replication capacity in macrophages. The majority of reisolates was capable of CD4-independent infection in NP-2/CCR5 cells and could replicate efficiently in macrophages. However, productive infection in NP-2 cells expressing CCR5 but not CD4 was in most cases revealed only after cocultivation of infected NP-2/CCR5 cells with hPBMC (CD4-independent-LOW). In comparison, infection of NP-2 cells expressing both CD4 and CCR5 resulted in more efficient syncytia induction and productive infection. Also the SMM-3 inoculum was capable of CD4-independent use of CCR5 and replicated well in macrophages. Reisolates obtained early in infection (2-week isolates from nine macaques and 3 or 4-month isolates from four macaques) showed a more pronounced CD4-independence (CD4-independent-HIGH) since virus production and/or syncytia induction could be detected directly in NP-2/CCR5 cells. Late isolates, especially from LTNP macaques, were more restricted in CD4-independent infections. This is in line with our previous study in which we showed that envelope clones from early SIVsm reisolates maintained a broad range of CD4-independence when the macaques were infected with a CD4-independent inoculum virus [27]. In the same study, CD4-dependence increased in late reisolates from macaques that developed neutralizing antibodies to the inoculum virus and to the early isolates.

Based on studies of CD4-independent laboratory-adapted variants of HIV-1, Hoffman and co-workers suggested that envelopes capable of binding to coreceptors in a CD4-independent manner are likely to have a "more open" conformation, similar to CD4-triggered CD4-dependent envelopes [17]. For SIV Chen *et al. *showed that the crystal structure of unligated envelope from CD4-independent SIVmac 32H needed to refold and move parts of the envelope 40 Å in order to reveal the structure of the coreceptor binding site known from the CD4-ligated HIV-1 HXBc2 envelope previously crystallized by Kwong *et al. *[36,37]. Many studies have shown an association between CD4-independence and neutralization sensitivity for HIV as well as SIV [21-26]. However, in these studies correlation between CD4-independence and neutralization sensitivity was based on fusion assays of pseudoviruses with expression of various cloned envelopes. In the present study, we used primary isolates that consist of a swarm of viruses expressing different envelopes and closer reflect the *in vivo *situation than cloned envelopes. We have previously shown with the material included in the present study that the 2-week isolates were neutralization sensitive while the 3 or 4-month isolates and especially late isolates evolved to neutralization resistance [30]. Likewise, CD4-independent use of CCR5 is more pronounced with virus isolates obtained shortly after infection of the macaques. Thus, newly infected macaques often harbored virus populations with a CD4-independent-HIGH and neutralization sensitive phenotype (Figure 5). At two weeks after infection the CD4-independent-HIGH phenotype was observed in isolates from approximately half of the P and SP macaques, while at the same time-point this was the only phenotype present in LTNP macaques. Gradual change to a CD4-independent-LOW and neutralization resistant virus population was evident and faster in progressors than LTNP macaques. This can be interpreted as a sign of a more open envelope conformation early as compared to late virus populations. Interestingly, in the P/SP group of macaques there was also a fraction of animals that late in infection had a CD4-independent-HIGH and neutralization resistant phenotype. Selection of CD4-independent variants has also previously been shown to occur in macaques with rapid progression of simian AIDS [28,29] and was characterized by absent or transient humoral and cellular immune responses [28]. Thus, it appears that CD4-independent use of CCR5 evolves in different directions in progressors and LTNP macaques. It is tempting to speculate that in LTNP macaques the overall immunity is more potently controlling the virus. Loss of immune control in progressors may lead to variants with a more CD4-independent and open conformation of the envelope.

CD4-independence of SIV envelopes have previously been correlated not only to neutralization sensitivity but to macrophage tropism as well [21,22]. Moreover, since macrophages have a lower CD4-expression than T lymphocytes it has been suggested that this would influence their susceptibility to HIV or SIV infection [38-41]. In the present study we found a clear correlation between CD4-independent use of CCR5 and macrophage tropism, inasmuch all isolates productively infected macrophages and also infected cells expressing CCR5 but not CD4 as shown by cocultivation of infected NP-2/CCR5 cells with hPBMC, infection of hPBMC with lysates and supernatants from infected NP-2/CCR5 cells. Thus, the majority of the SIVsm isolates were both macrophage-tropic and CD4-independent. However, the correlation between CD4-independence and macrophage-tropism was not strict since some of the isolates that appeared negative for CD4-independence even after cocultivation of NP-2/CCR5 and hPBMC could replicate to high levels in macrophages.

In infected MDM, virus particles can be observed within intra-cytoplasmic vesicles with characteristic multivesicular bodies known as late endosomes or major histocompability complex class II compartments [42,43]. Similarly and recently, reports have shown that large amounts of infectious virions accumulate in endosomal compartments within 293 T cells, in chronically infected human T lymphocytes [44]and as well as in primary macrophages [32]. Viruses accumulated intracellularly in macrophages can retain infectivity for at least 6 weeks shown by infection of MAGI cells with cell lysates from infected macrophages [32]. In the present study we found that in SIVsm-infected NP-2/CCR5 cells virus can mature intracellularly as shown by infection of hPBMC with cell lysates from infected cells. Virus production was also detected after contact of NP-2/CCR5 cells with hPBMC. It is tempting to speculate that the low or non-productive infection of non-CD4-expressing cells may be amplified by contact with T-lymphocytes *in vivo*. The importance of direct cell-to-cell spread in HIV infections is well known [45-47]. However, we also have to consider the possibility that the NP-2/CCR5 cell line is not infected and that viruses are only taken up by the NP-2/CCR5 cells in a fashion similar to that of DC-SIGN and transferred to PBMC after cocultivation [48,49]. Dentritic cells (DC) have been shown to capture and internalize extracellular HIV or SIV via DC-SIGN or DC-SIGNR and, without being infected, transmit virus to T cells *in trans*. In our system, we could show that infection of NP-2/CCR5 cells expressing CCR5 with CD4-independent-HIGH isolates resulted in a low level virus production seven days after infection and we could also observe syncytia induction in these cells. Interestingly, the compartment where HIV is captured by DC after DC-SIGN uptake has been shown to be similar to the multivesicular bodies where intracellular assembly and budding occurs in macrophages [50,51]. Transmission of HIV between cells has been shown to occur in an infectious or virological synapse that locally concentrates virus and receptors between infected and uninfected cells [52-55]. Ganesh *et al. *showed that viruses transferred by this synapse are poorly accessible to neutralizing antibodies [53]. Accordingly, when viruses assemble, bud and mature in endosomal compartments, this may provide means for cell to cell transfer of virus without encountering the immune system.

Mode of CCR5 receptor use may affect the virus-receptor interaction at entry. A high number of our SIVsm isolates could use all three chimeric CCR5-CXCR4 receptors included in this study. FC-1, with only the extracellular N-terminal exchanged for CXCR4 was the most commonly used chimera. This is in contrast with the results of Edinger *et al. *who showed that the N-terminal of CCR5 is the critical domain for CD4-independent entry of SIVsm envelope clones [56]. This group also found that the N-terminal of CCR5 in chimeric CCR2b/CCR5 chemokine receptors was sufficient for entry into CD4+ cells of the M-tropic strain SIVmac316 and, by contrast, the strains SIVmac239 and SIVmac251 required the presence of the second extracellular loop of CCR5 and exhibited a decreased dependence on the amino-terminal domain [8]. All the chimeric receptors tested in our present study expressed the extracellular loop 2 from CCR5, but in our hands, the N-terminal of CCR5 was usually not needed for infection. The SIVsm isolates, as well as SIVmac 251 and SIVmac 32H used the FC-1 receptor, with a CXCR4 N-terminal and CCR5 as second extracellular loop. This contrasts the use of chimeric coreceptors by primary HIV-1 R5-isolates that use FC-2 more often than FC-1. In fact FC-1 appeared to be the most restrictively used chimeric receptor by HIV-1 isolates [57,58]. In HIV-1 infection FC-4b using viruses emerge in patients who later switch viral phenotype from R5 to X4. In SIVsm infection, a surprisingly high number of the SIVsm reisolates infected cells expressing FC-4b. However, CXCR4-using SIVsm isolates were not more common among the isolates capable of using FC-4b compared to the total number of isolates. Our results indicate that SIVsm uses CCR5 in a different mode than HIV-1. HIV-1 isolates seem to be more restricted than SIV in the interaction with different chimeric coreceptors. The differences between HIV-1 and SIV are even more pronounced when viruses isolated on mPBMC are tested, since eight out of 11 mPBMC isolates could use all three chimeric coreceptors. It is possible that the SIV envelope is less dependent on conformational changes than the HIV-1 envelope and therefore less dependent on the exact structure of the coreceptor. It is also possible that the ability to use different variants of the CCR5 receptor can predispose the virus for CD4-independent entry. Again, viruses isolated on mPBMC were more CD4-independent than viruses isolated on hPBMC, indicating that human cells may select for CD4-dependence.

## Conclusion

Taken together, in this study we could show that CD4-independent infection of CCR5 expressing cells was a common characteristic of primary SIVsm isolates. Different phenotypes were observed among the virus isolates and in many cases CD4-independent infection was at a very low level and was only rescued after cocultivation of infected NP-2/CCR5 cells with hPBMC, defined as CD4-independent-LOW phenotype. Isolates obtained early in infection were often of CD4-independent-HIGH-phenotype. Changes towards a more CD4-dependet phenotype occurred over time; faster in macaques with a more progressive disease than in long-term non-progressors. While these changes were parallel with changes in sensitivity to neutralization (Figure 5) the ability to productively infect monocyte derived macrophages remained at steady high levels.

CD4-independent infections may conceivably have important consequences. First, cell and tissue tropism of SIVsm may broaden and lead to establishment of latently infected cell reservoirs in SIV infections. Second, intracellular maturation may provide the virus with a hideaway from the immune system, including the possibility of direct transfer of viruses between cells. One scenario has been reproduced by our experiments: the low or non-productive infection of CD4 negative cells was amplified by contact with PBMC. Our results also suggest that the SIV envelope is less dependent, than HIV-1, on the exact structure of the coreceptor compared to HIV-1 and that the ability to use different variants of the CCR5 coreceptor may influence CD4-independent entry. This difference may indicate that a higher dependence on CD4 for cell entry as well as a more specific binding to the coreceptor could have evolved in humans infected with HIV-1 and led to the more the pathogenic HIV-1 virus (first suggested by Edinger *et al. *in 1999 [59]).

## Methods

### Animals and disease progression

Female cynomolgus macaques (*Macaca fascicularis*) of Chinese origin were housed at the Swedish Institute for Infectious Disease Control. Housing and care procedures were in compliance with the general guidelines of the Swedish Animal Welfare Agency and all procedures were approved by the Local Ethical Committee on Animal Experiments. The SIVsm isolate SMM-3 (kindly provided by P. Fultz and H. McClure, Yerkes Regional Primate Research Center, Atlanta, GA, [60]) originated from a naturally infected sooty mangabey (*Cercocebus atys*) was used to infect thirteen macaques intravenously (IV) or intrarectally (IR) with 10 MID_50 _of cell free virus stocks produced in cultures of peripheral blood mononuclear cells (PBMC) from cynomolgus macaques [61]. The macaques were monitored for general clinical status. Blood samples for virus isolation, viral load and CD4+ T-cell count determinations were collected at regular intervals. The animals were kept until development of AIDS, or if asymptomatic, until the end of the study period, when they were euthanized. SIV RNA levels in plasma were measured using a highly sensitive quantitative competitive (QC) RT-PCR assay with a lower detection limit of 100 RNA equivalents/ml plasma, as described in details elsewhere [62]. The animals were monitored for changes in their CD4+ cell counts using two-color flow cytometric analysis as reported previously [63].

Based on the rate of disease progression, CD4 decline, time of death and, whenever available, viral load, the macaques were divided into three groups: progressor (P), slow progressor (SP) and long-term non-progressor (LTNP) [31]. Clinical status of the macaques are supplemented [see Additional file 1]. There was no difference in either the CD4 decline or viral load when comparing macaques inoculated by different routes of infection. As expected, decline of CD4+ T-cells was more pronounced in the first three months of infection, while decline was less thereafter. For the majority of animals the pattern of viral load was consistent with the observations by Ten Haaft *et al. *in that a threshold plasma virus load which was greater than 10^5 ^RNA equivalents/ml of plasma 6 to 12 weeks after inoculation could predict a faster disease progression [62]. In most of the P macaques plasma viral load was high initially (>10^6 ^RNA copies/ml) and stayed high [31]. The P macaques showed the fastest decline in CD4+ T-cell count and all animals developed simian AIDS or AIDS-related symptoms. Mean CD4 decline in the P group (seven animals) was -4.27% CD4+ T-cells/month (range -8.05 to -0.69% CD4+ T-cells/month). All P macaques were euthanized within 27 months post-infection and the mean time to AIDS in this group was 17 months. Virus isolation frequency was high (≥ 83%) with one exception (macaque C39: 58%). Macaques in the slow progressor (SP) group (two animals) did not show disease symptoms during the study period, however, the macaques showed a higher rate of CD4 cell decline and had a higher virus isolation frequency (-1.0% decline of CD4+ T- cells/month and virus isolation frequency ≥ 91%) than the macaques in the LTNP group (four animals, mean CD4+ T- cell decline/month -0.3% cells and mean virus isolation frequency 51%, range 10–72%). None of the four LTNP macaques evaluated in this study showed any signs of disease.

### Virus isolates

In the present study two to four isolates from each macaque were used depending on virus isolation frequency and the length of the macaque's survival time [see Additional file 1]. The virus isolates have previously been tested for coreceptor usage and neutralization sensitivity [30,31]. The first isolate was usually obtained as early as two weeks post infection, the second isolate by three or four months, the third isolate was chosen at a time in between the second and the last isolate and the last (fourth) isolate was collected by the time of euthanization. Virus isolation was performed by cocultivation of cynomolgus macaque PBMC (mPBMC) with mPBMC or with human PBMC (hPBMC) stimulated with phytophaemagglutinin (PHA-P) [64]. Due to the restricted availability of blood from macaques, limited numbers of isolates were available from cocultivation with mPBMC, thus the need for us to work with isolates obtained on hPBMC. Reisolates were passaged no more than two times. Cell-free supernatants were screened for the amount of reverse transcriptase (RT) with Cavidi HS-kit Lenti RT (Cavidi Tech, Sweden), and stored frozen at -80°C until used. SIVmac 32H and SIVmac 251 were used as reference virus [14].

### Infection of NP-2 and U87.CD4 cell lines

NP-2, a human glioma cell line, was engineered to stably express human CD4 and/or human CCR5, as described elsewhere [13,35,65]. Human glioma U87.CD4 cells, stably expressing CD4 and the chemokine receptors CCR5, or chimeric receptors between CCR5 and CXCR4 were previously described [2,33]. Chimeric receptors were constructed by replacing successively increasing portions of CCR5 with corresponding regions of CXCR4 by a modification of the single-overlap and extension PCR approach. The resulting three chimeric constructs and wild-type receptors were stably expressed in U87.CD4 cells. Parental U87.CD4 cells, engineered to express CD4 but no chemokine receptor, were also included in the experiments.

Cells in 500 μl of medium per well (without selective antibiotics) were seeded into 48-well plates 1 or 2 days prior to infection to obtain a 50%-confluent cell layer by the time of infection. For infection, medium was first removed, after which virus was added to duplicate wells in a volume of 350 μl/well. Cells were infected with virus stocks containing 2.7–3.5 log10 pg RT/well (median 3.2 log10 pg RT/well). After an overnight incubation, cells were washed with 1 ml PBS (0.12 M NaCl, 0.03 M phosphate [pH 7.2]), 1 ml of medium with polybrene (2 μg/ml) was added to each well, and the plates were further incubated. The cultures were kept for 7 days, and inspection for syncytia formation was performed daily. Supernatant culture fluids were collected at day 1 and 7 and production of RT was analyzed with Cavidi HS-kit Lenti RT (Cavidi Tech, Sweden). Supernatants were run undiluted and therefore values above 1000 pg RT/ml were over range. An infection was considered positive when RT activity at day 7 was at least twice the value of day 1, or alternatively positive when reaching above the threshold limit (50 pg RT/ml) when day 1 supernatant was below threshold.

### Cocultivation of NP-2/CCR5 with PBMC

PBMC from seronegative human blood donors were purified by Ficoll density gradient separation and were activated with 2.5 μg/ml of PHA-P for four days. Seven days after infection the NP-2/CCR5 cell cultures were washed with PBS and treated with EDTA-trypsine (2.5 mM EDTA and 0.125% trypsin) until detached from plastic. Hereafter all NP-2/CCR5 cells, from two parallel wells in the 48-well plates, were transferred to centrifuge tubes and washed once with PBS. The NP-2/CCR5 cells were further transferred to new wells in a 12-well plate and mixed with 6 × 10^5 ^PBMC/well from two PBMC donors in 2 ml RPMI medium. Supernatant culture fluids were collected after additional 6 days and production of RT was quantified with Cavidi HS-kit Lenti RT (Cavidi Tech, Sweden).

In an additional experiment NP-2/CCR5 cells were infected with virus isolates in parallel wells as described above. The infected cells from two parallel wells were lysed and the lysates were titrated on hPBMC in order to detect infectious virus in the cytoplasm. For cell-lysis, we adopted a method previously described by Sharova *et al. *where infectious virus was recovered directly from cytoplasmic lysates of macrophages [32]. Briefly, cultures from each well were first washed with 200 μl PBS and added 200 μl trypsin (0.25%) to exclude possible virus attached on the outer cell membrane and to detach the cells from the plastic. Another washing step with PBS was followed after which the cells were incubated with 200 μl 0.001% Triton X-100 in PBS for 5 minutes and put in -80°C for complete freezing. Cells where then thawed and raptured by mechanical force by extensive pipetting and complete lysis was observed by microscope analysis after staining of cells with tryptan blue. Infections with lysed cell material in three five-fold dilution steps were performed on 10^5 ^PBMC/well in 96-well plates and cells were mixed from four human donors. Infected cultures were incubated over night (37°C in 5% CO_2 _atmosphere) in RPMI medium with 10% FBS, antibiotics and 2 μg/ml polybrene at a total volume of 200 μl. Infections with NP-2/CCR5 cell lysates were carried out. Next day, cultures were washed with PBS and further incubated with fresh medium. Supernatant culture fluids were collected at day 1 and 7 and production of RT was analyzed with Cavidi HS-kit Lenti RT (Cavidi Tech, Sweden). The level of detection for RT production was 50 pg/ml.

### Isolation and infection of monocyte-derived macrophages (MDM)

MDM were isolated from PBMC from blood donors or cynomolgus macaques according to a previously established protocol [66]. These experiments involved seven different human MDM donors and every isolate was tested on MDM from 2 to 4 blood donors in independent experiments. After Ficoll separation, 15 × 10^5 ^cells/cm^2 ^were seeded in 24- or 48-well plates in RPMI medium supplemented with antibiotics, 20% FBS and 10% human serum (HS), pooled from nine different normal donors. Cells were cultured at 37°C in 5% CO_2 _atmosphere. Six days after seeding, non-adherent cells were removed by extensive rinsing with PBS. In addition, cultures were treated with trypsin to further remove non-macrophage cells. Adherent cells were maintained in medium without HS. Cytochemical staining for non-specific esterase (Sigma, Germany) at day 7 showed over 98% positive cells.

After seven days the MDM cultures were infected with the virus isolates. Prior to infection, cells were washed twice with PBS and fresh medium containing 2 μg/ml polybrene was added to the wells. Macrophages were infected with virus stocks containing 3.0–3.1 log10 pg RT/well (in 88% of the cultures). Sequential isolates from each monkey were tested simultaneously. The day after infection, cultures were rinsed twice with PBS and fresh medium was added. In order to compare the kinetics of virus replication in MDM 20 SIVsm virus isolates were tested on MDM from four different human blood donors in a pilot study. Supernatants of infected MDM were analyzed for virus production by testing reverse transcriptase activity (Cavidi HS-kit Lenti RT) at day 1, 3, 7, 11 and 15 after infection. These experiments with peak replication at day 15 or beginning of steady state replication at day 11 after infection, allowed us to choose day 15 for comparison of macrophage tropism of viruses in following experiments (data not shown).

### Plaque reduction neutralization assay

The GHOST(3) cell line-based plaque assay is a single cycle infectivity assay for HIV and SIV, where green fluorescent protein (GFP) expression is a hallmark of infection [67]. The assay was performed in 96-well microtiter plates where infected single cells or syncytia appear as distinct green fluorescent plaques and are counted as plaque-forming units (PFU). The serum (H55:16) used in the study was obtained from an infected monkey that remained asymptomatic and had high neutralizing titers towards SIVsm [30]. For the neutralization assay, virus and heat inactivated sera were diluted and mixed in culture medium, to give 20 to 100 PFU/well and 1:20 serum dilution. The virus and serum mixtures were incubated at 37°C for one hour, followed by further dilution in two 5-fold steps, and distributed to triplicate wells. The day after infection the virus-serum mixtures were replaced with fresh medium. The cultures were checked for expression of GFP using fluorescence microscopy three days after infection and virus titers were calculated as PFU/ml: (average number of plaques in triplicate wells × virus dilution)/volume in the well. The neutralizing property of the serum was calculated as percentage plaque reduction of the virus titration by the formula 1-(PFU with serum/PFU without serum) × 100. Based on intra-assay variation, the cut-off for neutralization was set to 30% (≥ 3 × SD), that is, values below 30% are considered as negative for neutralization.

### Statistic analysis

McNemar's test was used to analyze if the proportions of CD4-independent phenotypes of paired early and late isolates are the same.

## Competing interests

The author(s) declare that they have no competing interests.

## Authors' contributions

AL planned the experiments, carried out cell assays and wrote the manuscript. EV was involved in design of the experiments and carried out cell assays. HH participated in the study design, helped in drafting the manuscript. RT was responsible for the animal studies and helped designing the experiments. EMF conceived of the study and participated in its design and coordination and helped to write the manuscript. All authors read and approved the final manuscript.

## Supplementary Material

Additional file 1 Table over features of the macaques and virus isolates used in this study. This table summarise disease progression of the macaques included in the study as well as the phenotypic characteristics of the virus isolates from the animals. Click here for file
